# Varicose veins and venous thromboembolism in inflammatory rheumatic diseases: vascular complications and rehabilitation approaches

**DOI:** 10.1007/s00296-026-06070-y

**Published:** 2026-02-02

**Authors:** Olena Zimba, Mariusz Korkosz, Anvar Sultanov, Bekzhan A. Permenov, Burhan Fatih Kocyigit

**Affiliations:** 1 Department of Rheumatology, Immunology and Internal Medicine, University Hospital in Kraków, Kraków, Poland; 2 National Institute of Geriatrics, Rheumatology and Rehabilitation, Warsaw, Poland; 3 Department of Internal Medicine N2, Danylo Halytsky Lviv National Medical University, Lviv, Ukraine; 4 Department of Rheumatology and Immunology, Jagiellonian University Medical College, Kraków, Poland; 5 Department of Social Health Insurance and Public Health, South Kazakhstan Medical Academy, Shymkent, Kazakhstan; 6 Clinical Diagnostics Center, Khoja Akhmet Yassawi International Kazakh-Turkish University, Turkistan, Kazakhstan; 7 Department of Cardiac Surgery Anesthesiology and Intensive Care, Heart Center Shymkent, Shymkent, Kazakhstan; 8 Department of Internal Medicine, Khoja Akhmet Yassawi International Kazakh-Turkish University, Turkistan, Kazakhstan; 9 Department of Physical Medicine and Rehabilitation, University of Health Sciences, Adana Health Practice and Research Center, Adana, Türkiye Turkey

**Keywords:** Rheumatic diseases, Arthritis, Venous thromboembolism, Venous thrombosis, Varicose veins, Rehabilitation

## Abstract

Inflammatory rheumatic diseases (IRDs) compromise vascular integrity through systemic inflammation and (auto) immune reactions, which are associated with an increased risk of vascular complications. Venous stasis, endothelial dysfunction, and coagulation imbalance are the primary pathophysiological factors behind thrombotic events in these individuals. The incidence of venous thromboembolism (VTE) in IRDs is higher than in the general population, although the magnitude of this increase varies across diseases. Inflammatory damage to vessel walls, diminished elasticity, and compromised muscle pump function may contribute to the formation of varicose veins (VVs). A sedentary lifestyle, decreased muscular strength, and weight gain worsen the condition, particularly by adversely affecting venous return in the lower extremities. Consequently, the prevention of vascular issues in IRDs should be facilitated by both pharmaceutical interventions and rehabilitation with lifestyle modifications. A multidisciplinary rehabilitation strategy—encompassing regular physical activity, compression therapy, inflammation management, weight control, and patient education—enhances venous return, mitigates thrombosis risk, and improves quality of life.

## Introduction

Varicose veins (VVs) are defined as dilated and tortuous veins according to the Medical Subject Headings (MeSH) vocabulary [1]. The leading cause of this condition is venous blood stasis resulting from valve deterioration and impaired function [2]. VVs are a chronic vascular disease affecting the superficial veins of the lower limbs [3].

MeSH defines venous thromboembolism (VTE) as a condition marked by the obstruction of one or more veins due to a clot (thrombus) in the circulatory system [4]. VTE encompasses the triad of venous stasis, endothelial injury, and hypercoagulability (Virchow’s triad), and these pathophysiological mechanisms result in considerable mortality and morbidity, particularly when associated with comorbidities [5]. Inflammation is an important trigger that facilitates the development of venous stasis, endothelial damage, and hypercoagulability within the context of Virchow’s triad.

VVs are among the most frequent vascular disorders in the general population. Epidemiological studies indicate that prevalence rates reach 40% [6]. The prevalence is higher in females, attributable to hormonal changes, pregnancy, and lifestyle factors [7]. On the other hand, VTE, although less common, has more severe clinical repercussions. The annual incidence of VTE in the general population is around 1–2 cases per 1,000 individuals and escalates markedly due to older age, obesity, immobility, malignancy, surgical interventions, and hereditary coagulation disorders [8].

**Fig. 1:**
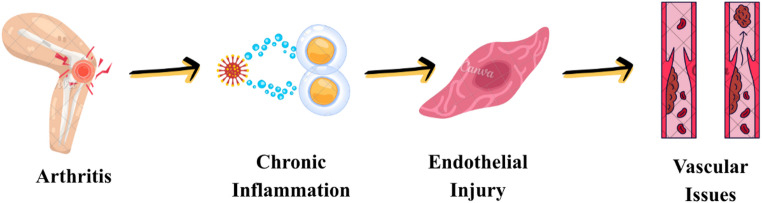
Chronic inflammation, endothelial activation, and cytokine-mediated prothrombotic state in rheumatic diseases

Inflammatory rheumatic diseases (IRDs) are marked by persistent inflammation and immune-mediated vascular injury. The persistent inflammatory process, endothelial impairment, and microvascular injury contribute to the development of VVs and VTE in IRDs (Fig. 1). Chronic inflammation stimulates endothelial cells, establishing a procoagulant milieu that heightens the propensity for thrombosis and contributes to the structural compromise of the vascular wall, which may be involved in the development of VVs. Persistent endothelial stimulation induces extracellular matrix remodeling, elastic fiber degradation, and smooth muscle cell dysfunction, thereby reducing venous compliance and promoting progressive venous dilatation. These structural changes impair venous valve function, leading to venous reflux [9, 10]. The development of VVs and VTE in IRDs is linked not only to the disease but also to various confounding factors. Age and sex represent crucial demographic variables within this context. Excess body weight and physical inactivity contribute to venous stasis by diminishing venous return, thereby increasing the risk of VVs and thrombosis [11]. In IRDs, calf muscle pump dysfunction is a consequence of chronic inflammation-related pain, joint stiffness, muscle weakness, fatigue, and decreased physical activity, rather than a direct inflammatory involvement of skeletal muscle. It plays an essential role in promoting venous stasis.

VVs and VTE are regarded as both localized vascular abnormalities and clinical indicators of systemic inflammation in IRDs. Understanding the links between these two vascular disorders and IRDs is essential for prompt identification of issues and for formulating preventive management strategies. 

### Aim

This review aims to thoroughly examine the pathophysiological mechanisms, epidemiological data, and clinical outcomes related to the development of VTE and VVs in IRDs. The importance of risk factors, including obesity, physical inactivity, and disease activity, is highlighted, and the current literature on diagnosis, prevention, and rehabilitation strategies is overviewed. 

### Search strategy

Prior to initiating the literature search, a detailed search plan was established, and the methodology was structured in alignment with the recommendations by Gasparyan et al. [12].

The combinations of keywords utilized in this directive are organized in the following manner: ‘Venous Thromboembolism and Rheumatology’ or ‘Venous Thromboembolism and Rheumatic Diseases’ or ‘Venous Thromboembolism and Arthritis’ or ‘Venous Thrombosis and Rheumatology’ or ‘Venous Thrombosis and Rheumatic Diseases’ or ‘Venous Thrombosis and Arthritis’ or ‘Varicose Veins and Rheumatology’ or ‘Varicose Veins and Rheumatic Diseases’ or ‘Varicose Veins and Arthritis’.

The search was performed utilizing global databases, including Scopus, Web of Science, PubMed/MEDLINE, and the Directory of Open Access Journals (DOAJ). The inclusion criteria encompassed studies that investigated clinical or epidemiological findings related to VTE and VVs in adults with IRDs. No specific article type was designated as an exclusion criterion, and no limitations were applied concerning the publication year. The review was confined to articles published in English.

The references of the included articles were thoroughly examined, and additional studies pertinent to the topic were identified and incorporated into the analysis.

### Rheumatoid arthritis

Rheumatoid arthritis (RA), a chronic inflammatory disorder, causes sustained synovial tissue inflammation. This process affects both the joints and the vascular system, leading to endothelial dysfunction and an increased tendency towards thrombosis [13]. Cytokines and immune complexes induce endothelial dysfunction, enhancing vascular permeability, upregulating adhesion molecules, and activating platelets. Consequently, procoagulant substances are secreted in greater amounts, natural anticoagulant mechanisms are inhibited, and vascular tone is compromised by oxidative stress. As a result, the coagulation system stays perpetually active, establishing a conducive environment for venous thrombosis [14].

A systematic review revealed a significantly increased risk of VTE in patients with RA [15]. In this analysis, the relative risks for deep vein thrombosis (DVT) were 2.08 (95% Confidence Interval [CI]: 1.75–2.47), for pulmonary embolism (PE) 2.17 (95% CI: 2.05–2.31), and for total VTE 1.96 (95% CI: 1.81–2.11) in RA patients. A more recent meta-analysis found a 2.25 (95% CI: 1.70–2.98) increased risk for DVT, 2.15 (95% CI: 1.39–3.49) for PE, and 2.23 (95% CI: 1.79–2.77) for total VTE in patients with RA [16].

In a cohort study based on Swedish national registry data, the 1-year cumulative VTE incidence was 0.71% in RA patients and 0.36% in the general population [17]. These data corresponded to a 1.88-fold increased risk for RA (95% CI: 1.65–2.15) [17]. The risk of VTE in RA patients increased significantly with disease activity. While the incidence of VTE was 0.52% in remission, it increased to 1.08% in high disease activity [17]. The sedentary lifestyle and diminished muscle strength in patients with RA adversely affect venous return to the lower extremities, thereby promoting the onset of VV [2, 18].

### Spondyloarthritis

Spondyloarthritis (SpA) is a group of chronic inflammatory disorders comprising several subtypes affecting the axial skeleton and peripheral joints. In SpA, systemic inflammation and activation of the TNF-α, IL-17/IL-23 axis enhance all aspects of Virchow’s triad by elevating endothelial tissue factor expression and promoting platelet-endothelial interactions. This process increases the risk of venous thrombosis, especially in disease flares and in comorbidities [9, 19].

In an extensive population-based cohort study, the risk of VTE was evaluated in patients with ankylosing spondylitis (AS) [20]. The incidences of PE, DVT, and total VTE were 0.79, 1.06, and 1.56/1000 person-years in AS patients, respectively, compared to 0.40, 0.50, and 0.77/1000 person-years in the control group [20]. After adjusting for all variables, the hazard ratio (HR) values ​​were 1.36 (95% CI: 0.92–1.99) for PE, 1.62 (95% CI: 1.16–2.26) for DVT, and 1.53 (95% CI: 1.16–2.01) for VTE [20].

A cohort study examined the risk of major cardiovascular events, including VTE, in individuals with AS, psoriatic arthritis (PsA), and undifferentiated SpA (uSpA) relative to the general population [21]. The standardized VTE incidence was 3.6/1000 person-years in AS, 3.2/1000 person-years in PsA, and 3.5/1000 person-years in uSpA, and 2.2/1000 person-years in the control group [21]. VTE risk was found to be roughly 50% greater in all SpA subtypes, with age- and sex-adjusted HRs of 1.53 (95% CI: 1.25–1.87) for AS, 1.55 (95% CI: 1.29–1.86) for PsA, and 1.55 (95% CI: 1.19–2.02) for uSpA [21]. Data from the Israeli health database indicated a significant elevation in the risk of VTE among individuals with PsA in univariate analysis (HR: 1.40; 95% CI: 1.05–1.87) [22].

Regarding VV, the same concept applies to SpA, in which chronic inflammation affects the venous wall and valve apparatus, leading to diminished elasticity, dilation, and valve insufficiency. In PsA, the extensive inflammatory environment linked with concurrent skin and nail manifestations undermines microvascular integrity, whereas in AS, postural constraints and reduced physical activity negatively affect venous return in the lower extremities. Consequently, sedentary lifestyle, along with diminished muscular power and weight gain, promotes the onset of varicose veins [10, 23].

### Systemic lupus erythematosus

Systemic lupus erythematosus (SLE) is a chronic inflammatory autoimmune disease characterized by multisystem involvement. A prominent consequence of inflammation in SLE is endothelial dysfunction and an increased tendency for thrombosis. Inflammation induces endothelial activation and injury via autoantibody expression and immune complex accumulation, promoting the onset of VTE. Multiple studies indicate that the risk of VTE is markedly elevated in SLE relative to the general population. This risk correlates with both disease activity and the existence of antiphospholipid antibodies [24]. The risk of thrombosis escalates exponentially with antiphospholipid antibody positivity [24–26].

A systematic review found a 4.38-fold greater risk of VTE in SLE patients compared with the general population (RR 4.38; 95% CI: 2.63–7.29) [27]. Subanalyses indicated a 6.35-fold elevation in the risk of DVT (95% CI: 2.70–14.94) and a 4.94-fold elevation in the risk of PE (95% CI: 1.90–12.86) [27]. The risk was significantly increased in the presence of antiphospholipid antibody positivity or antiphospholipid syndrome [27].

In a cohort study from the Western Australian health database, 12.8% of SLE patients suffered VTE (3.3% in controls), resulting in a 4.26-fold higher risk (95% CI: 3.60–5.05) [28]. The strongest risk factors were antiphospholipid antibody positivity, serositis, lupus nephritis, and thrombocytopenia [28]. Furthermore, VV was found to be an independent risk factor [28].

In a study based on the Taiwan National Health Insurance database, the risk of DVT was 12.8-fold (95% CI: 9.1–18.1) and the risk of PE was 19.7-fold (95% CI: 11.9–32.8) increased in SLE patients [29]. The incidence rates were documented as 15.1 per 10,000 person-years for DVT and 10.2 per 10,000 person-years for PE [29].

Chronic inflammation and autoimmune processes in SLE markedly increase the risk of VTE by inducing endothelial injury and coagulation activation. Consequently, early evaluation of thrombosis risk, screening for antiphospholipid antibodies, and tailored preventative strategies in patients with SLE are essential.

### Systemic sclerosis

Systemic sclerosis (SSc) is a chronic connective tissue disorder marked by extensive fibrosis, vascular dysfunction, and autoimmune inflammation. Endothelial damage and microvascular disruption are primary pathophysiological hallmarks, leading to organ fibrosis and thrombotic events in SSc [30].

In a cohort study on SSc patients enrolled in the Toronto Scleroderma Program, VTE developed at a rate of 3.4% [31]. Independent risk factors included pulmonary arterial hypertension (OR: 3.77; 95% CI: 1.83–8.17), peripheral arterial disease (OR: 5.31; 95% CI: 1.99–12.92), Scl-70 positivity (OR: 2.45; 95% CI: 1.07–5.30), and the presence of anticardiolipin antibodies (OR: 5.70; 95% CI: 1.16–21.17) [31].

A recent meta-analysis indicated that the overall risk of VTE in SSc patients increased by 2.37-fold (95% CI: 1.61–3.5) [32]. Subanalyses revealed a 3.15-fold increase in the risk of PE (95% CI: 1.32–7.54) and a 5.19-fold increase in the risk of DVT (95% CI: 1.51–17.01) [32].

In a cohort study, the incidence of PE was 3.47/1000 person-years, DVT was 3.48/1000 person-years, and total VTE was 6.56/1000 person-years in the SSc group [33]. In the control group, the rates were 0.78, 0.76, and 1.37 per 1000 person-years, respectively [33]. Multivariate analysis revealed an HR of 3.73 (95% CI: 1.98–7.04) for PE, 2.96 (95% CI: 1.54–5.69) for DVT, and 3.47 (95% CI: 2.14–5.64) for total VTE in SSc patients [33]. 

VV in SSc may arise through processes similar to those underlying chronic venous insufficiency. Immobility, diminished muscle pump efficacy due to fibrotic alterations in the skin, and increased circulatory viscosity may facilitate the onset of venous varicosities by impeding venous return in the lower extremities. Moreover, endothelial dysfunction and reduced elastic fiber content compromise the integrity of venous walls [34, 35].

In SSc, endothelial damage, microvascular dysfunction, and hypercoagulability interact to establish a significant pathophysiological foundation for the occurrence of VTE and VV. 

### Sjögren syndrome

Sjögren Syndrome (SS) is a chronic autoimmune disorder with systemic consequences that is distinguished by lymphocytic infiltration of the exocrine glands. While salivary and lacrimal gland involvement is the most visible clinical sign, the disorder also causes systemic vascular inflammation, endothelial dysfunction, and an increased risk of thrombosis [36].

A nationwide cohort study in Taiwan reported an incidence of DVT of 5.81 per 10,000 person-years in the SS group and 3.11 per 10,000 person-years in the control group, whereas the incidence of PE was 6.43 and 1.97 per 10,000 person-years, respectively [37]. A multivariate study indicated HRs of 1.83 (95% CI: 1.16–2.89) for DVT and 3.29 (95% CI: 2.03–5.31) for PE in individuals with SS [37].

In another cohort study, the incidence of PE was 3.85 per 1000 person-years, the incidence of DVT was 2.75 per 1000 person-years, and the overall rate of VTE was 5.24 per 1000 person-years in the SS group, in contrast to 0.89, 0.79, and 1.44 per 1000 person-years in the control group, respectively [38]. Multivariate analysis indicated a markedly elevated risk in SS patients: HR = 4.07 (95% CI 2.04–8.09) for PE, HR = 2.80 (95% CI 1.27–6.17) for DVT, and HR = 2.92 (95% CI 1.66–5.16) for total VTE [38].

A meta-analysis indicated that the risk of VTE was 2.05 times greater than in the general population (RR: 2.05; 95% CI: 1.86–2.27) [39].

### Crystal arthropathies

Crystal Arthropathies induce not only local joint inflammation but also a severe systemic inflammatory and vascular response. In gout, elevated disease activity was significantly correlated with enhanced thrombin production [40].

A national cohort study in Taiwan reported an incidence of DVT of 13.48 per 10,000 person-years in the gout group and 9.77 per 10,000 person-years in the control group [41]. Gout elevated the risk of DVT by 38% (HR: 1.38; 95% CI: 1.18–1.62). This risk persisted after excluding comorbidities and conventional risk factors [41].

The risk of VTE was investigated both before and after gout diagnosis in a population-based cohort study [42]. Patients with gout had a HR of 1.22 (95% CI: 1.13–1.32) for VTE, 1.28 (95% CI: 1.17–1.41) for DVT, and 1.16 (95% CI: 1.05–1.29) for PE [42].

A meta-analysis indicated a 33% increase in the risk of venous VTE in gout (HR: 1.33; 95% CI: 1.21–1.46) [43]. Subanalyses showed a 40% elevation in the risk of DVT (HR: 1.40; 95% CI: 1.22–1.62) and an 18% rise in the risk of PE (HR: 1.18; 95% CI: 1.07–1.3) [43].

### Inflammatory myopathies

Inflammatory myopathies are a group of autoimmune diseases characterized by systemic inflammation affecting skeletal muscles. These diseases are characterized not only by muscle weakness but also by widespread vascular involvement and coagulation activation [44].

A retrospective cohort study on 1144 patients with inflammatory myopathy revealed a thromboembolic incident rate of 2.1% (*n* = 24) [45]. 

In a Swedish population-based cohort study, 6.6% (*n* = 28) of patients with inflammatory myopathy and 2% (*n* = 48) of the control group experienced a first VTE [46]. The HR for VTE was 7.81 (95% CI: 4.74–12.85) [46].

In a study by Notarnicola et al. [47], 246 patients with idiopathic inflammatory myopathy were assessed, with arterial or venous thrombotic events observed in 21% (*n* = 51) of cases. The thrombotic process was independently associated with age (OR 1.04; 95% CI 1.01–1.06; *p* = 0.004) [47]. 

A meta-analysis revealed that the risk of VTE is 4.36-fold greater in individuals with inflammatory myopathy (RR: 4.364; 95% CI: 2.13–8.95) [48]. Subanalyses indicated that the risk of PE was 3.50 times greater (95% CI: 2.86–4.3), while the risk of DVT was 7.48 times greater (95% CI: 3.36–16.68) [48].

### Vasculitis

Vasculitis is a clinically heterogeneous category of IRDs. Endothelial injury due to immune complex accumulation and complement activation, heightened tissue factor expression, and platelet activation provoke the coagulation system, increasing the risk of VTE in vasculitides [49, 50].

A meta-analysis revealed a significantly elevated risk of VTE across all vasculitis subtypes [51]. The risk was approximately 3.9-fold (RR: 3.94; 95% CI: 1.11–14.01) in patients with granulomatosis with polyangiitis, 3-fold (RR: 3; 95% CI: 2.2–4.09) in patients with polyarteritis nodosa, and 2.3-fold (RR: 2.26; 95% CI: 1.38–3.71) in patients with giant cell arteritis [51].

Aviña-Zubieta et al. [52] conducted a population-based cohort analysis revealing an incidence of VTE at 13.3 per 1000 person-years, PE at 7.7 per 1000 person-years, and DVT at 8.5 per 1000 person-years within the giant cell arteritis cohort. Multivariate analysis revealed a HR of 2.49 (95% CI: 1.45–4.3) for VTE, a HR of 2.71 (95% CI: 1.32–5.56) for PE, and a HR of 2.78 (95% CI: 1.39–5.54) for DVT [52]. The frequency of venous thrombosis in patients with Behçet disease was significantly higher than that in the control group, as demonstrated by a study conducted by Ames et al. [53].

A retrospective cohort study revealed that the incidence of coronary events in patients with antineutrophil cytoplasmic antibody-associated vasculitis was 15 times greater than that observed in the general population [54]. Additionally, the stroke incidence was found to be 11 times higher, while the rate of VTE was 20 times higher [54].

### Prevention and diagnosis of varicose veins and venous thromboembolism in inflammatory rheumatic diseases

The primary strategy for preventing vascular issues in IRDs is the prompt recognition and consistent monitoring of risk factors. Chronic inflammation, endothelial dysfunction, oxidative stress, and physical inactivity compromise vascular integrity in these individuals, elevating the risk of thrombosis and venous insufficiency. Obesity is the prominent modifiable comorbid condition. Elevated intra-abdominal pressure, diminished venous return, a sedentary lifestyle, and low-grade chronic inflammation induced by adipose tissue contribute to the development of both VVs and VTE [55, 56].

Weight management, regular exercise, and balanced dietary programs should be prioritized. Moreover, physiotherapy programs that facilitate patient mobility, manage disease activity, promote smoking cessation, and prevent extended immobilization are beneficial preventative interventions. Additional methods that are effective in preventing vascular complications include [57–59]:

- Regular mobilization and physiotherapy to enhance venous return and diminish stasis, particularly in individuals with restricted mobility or joint deformities;
- Compression therapy and elastic stockings to enhance venous circulation in cases of lower extremity edema or venous insufficiency;
- Prophylactic pharmacological therapies for individuals exhibiting elevated disease activity or a history of thrombosis during hospitalization, surgical procedures, or flares;
- Ceasing tobacco use, maintaining adequate hydration, avoiding prolonged sitting or standing, and engaging in regular leg exercises.

The initial step in assessing VVs is a comprehensive clinical evaluation. Patients frequently experience heaviness in the legs, discomfort, evening exacerbation of edema, cramping, and prominence of superficial veins. The physical examination reveals alterations in skin pigmentation, edema, and prominent varicose veins [60]. Doppler ultrasonography is the established technique for evaluation. This noninvasive imaging modality can evaluate venous flow direction, valve function, reflux, and the likelihood of thrombosis. Moreover, the patency of the deep venous system and its association with the superficial veins can be comprehensively illustrated [61].

Clinicians should retain a heightened degree of suspicion for VTE in patients with IRDs. Thrombosis should be considered, especially when symptoms including leg discomfort, edema, unexplained dyspnea, or chest pain are present. D-dimer testing is an initial screening test. Ultrasonography for DVT and computed tomography pulmonary angiography for PE are established diagnostic techniques [62, 63].

### Rehabilitation of patients with varicose veins and venous thromboembolism in inflammatory rheumatic diseases

Rehabilitation of patients with VV and VTE should include not only physical recovery, but also circulatory restoration and thrombosis risk reduction. The main objectives of rehabilitation include enhancing venous return, minimizing edema, improving muscle pump function, and reinstating the patient’s overall physical capacity. Muscle weakness, joint deformities, and pain often lead to physical inactivity in individuals with IRDs, worsening venous stasis and elevating the risk of thrombosis. Consequently, mobilization stands out as the crucial element of rehabilitation [64–66]. Well-structured rehabilitation programs not only improve muscular strength and joint range of motion but also reduce stasis by increasing venous return [67–70]:

- Low-intensity aerobic exercise (e.g., walking, cycling, aquatic therapy);
- Elevating the legs and performing passive or active joint movements;
- Resistance workouts strengthening the quadriceps and calf muscles;
- Physiotherapy and hydrotherapy alleviating joint pain and enhancing adherence to exercise regimens.

Compression therapy is an important component of the rehabilitation process that enhances venous return and mitigates venous stasis in IRDs. Graduated elastic compression stockings reduce venous pressure by establishing a pressure gradient in the lower limbs, thereby decreasing valvular insufficiency and minimizing edema. Pneumatic compression devices serve as an effective adjunct therapy to enhance venous circulation, particularly in those with restricted mobility. Combined with pharmacological therapies, compression therapy provides a synergistic benefit by reducing venous stasis [58, 71].

The management of vascular issues in IRDs should extend beyond physical rehabilitation to include pharmacological intervention and multidisciplinary teamwork. In patients with a heightened risk of thrombosis, especially during immobility, surgical procedures, or active inflammation, pharmacological interventions should be administered with physiotherapy. This approach prevents venous stasis and diminishes the likelihood of thrombus development. Moreover, effective collaboration among rheumatology, cardiology, and physical therapy/rehabilitation teams is essential to safeguard the vascular system and manage systemic inflammation. This comprehensive strategy seeks to avoid thrombosis while promoting sustained functional recovery in rheumatic patients [72, 73].

Patient education programs should explicitly and thoroughly explain the disease’s causes, lifestyle modifications, and the significance of treatment compliance. Enhancing awareness, especially regarding the establishment of consistent exercise routines, smoking cessation, sufficient hydration, and avoidance of prolonged inactivity, reduces the risk of thrombosis and improves overall quality of life. Group education and personalized counseling sessions strengthen patient involvement in the management (Table 1) [74].

**Table 1:** Core components of rehabilitation in inflammatory rheumatic diseases

Component	Primary Goal
Physical Rehabilitation	Activate the calf muscle pump to improve venous return and prevent stasis.
Compression Therapy	Reduce venous pressure, limit reflux, and prevent edema formation.
Patient Education	Promote adherence to lifestyle modifications, exercise, and preventive care.
Multidisciplinary Approach	Integrate rheumatology, cardiology, and physical rehabilitation teams for comprehensive vascular care.
Psychosocial Support	Improve motivation, treatment adherence, and overall quality of life.

### Perspectives

Management of VTE and VVs in IRDs should not be confined to treating existing complications; it should be complemented by tailored rehabilitation. In the future, biomarker-based risk assessment models and sophisticated imaging techniques (such as ultrasound and MRI-based technologies that dynamically monitor vascular endothelial function) will aid in early detection of thrombosis risk.

Artificial intelligence-powered clinical decision systems and digital health applications have the potential to improve thrombosis prevention in IRDs by analyzing individual risk profiles and monitoring factors like exercise, medication adherence, and nutritional habits in real time. Telemedicine-based exercise regimens and home compression/mobilization tracking devices may enhance rehabilitation continuity, particularly for patients with restricted mobility.

Future research is anticipated to provide deeper insights into the mechanisms of endothelial damage and the interactions between the coagulation and immune systems, specific to each type of IRD. This will facilitate the customization of both thromboprophylaxis and rehabilitation protocols tailored to individual patients. 

## Conclusion

VVs and VTE represent vascular manifestations that may occur in the context of chronic inflammation in IRDs. The combination of chronic inflammation, venous stasis, endothelial dysfunction, and hypercoagulability is associated with an increased susceptibility to thrombosis and venous insufficiency in these individuals. Chronic inflammation and reduced physical activity may play a role in venous dysfunction associated with VVs. Therefore, the care of IRDs should be viewed as a comprehensive approach that incorporates early diagnosis, multidisciplinary monitoring, lifestyle modifications, rehabilitation, and pharmacological interventions. Managing obesity, engaging in regular exercise, using compression therapy, and effectively addressing inflammation are essential for minimizing vascular issues.
